# Research Progress on Characterization Techniques for the Corrosion Behavior of Bronze Artifacts

**DOI:** 10.3390/ma19010162

**Published:** 2026-01-02

**Authors:** Hongliang Li, Yongdi Zhao, Xiaohui Wang, Hanjie Guo, Chao Ren, Chunyan Liu, Li Xiang

**Affiliations:** 1 School of Metallurgical and Ecological Engineering, University of Science and Technology Beijing, Xueyuan Road 30, Beijing 100083, China; lihongliang@ustb.edu.cn (H.L.); guohanjie@ustb.edu.cn (H.G.); 2 School of Materials Science and Engineering, University of Science and Technology Beijing, Beijing 100083, China; d202310305@xs.ustb.edu.cn; 3 State Key Laboratory for Advanced Metals and Materials, University of Science and Technology Beijing, Beijing 100083, China; wxhui@ustb.edu.cn; 4 School of Materials Engineering, Shanxi College of Technology, Shuozhou 036000, China; 5 Qingdao Yian Construction Limited Company, Qingdao 266109, China

**Keywords:** bronze artifacts, corrosion, X-ray fluorescence, X-ray computed tomography, neutron tomography

## Abstract

Ancient bronzes are invaluable for studying the cultures and history of ancient societies around the world. However, corrosion can diminish their research and aesthetic value, as well as affect their longevity. Therefore, it is crucial to study the corrosion behavior and mechanisms of these artifacts using advanced characterization techniques. This article provides a systematic review of the corrosion behavior of bronze artifacts and the advanced characterization techniques employed in their study. It summarizes the corrosion mechanisms of bronze artifacts and the factors affecting corrosion, including composition, structure, and the external environment. It also describes advanced analytical techniques for characterizing corrosion products and mechanisms, such as X-ray fluorescence (XRF), laser ablation coupled to quadrupole mass spectrometry (LAMQS), X-ray tomography (CT), and neutron diffraction. Bronze corrosion studies can be enhanced by the integration of artificial intelligence (AI) and machine learning (ML). Finally, it discusses potential future research directions in the field of bronze artifact corrosion and conservation.

## 1. Ancient Bronze Artifacts

**Figure 1:**
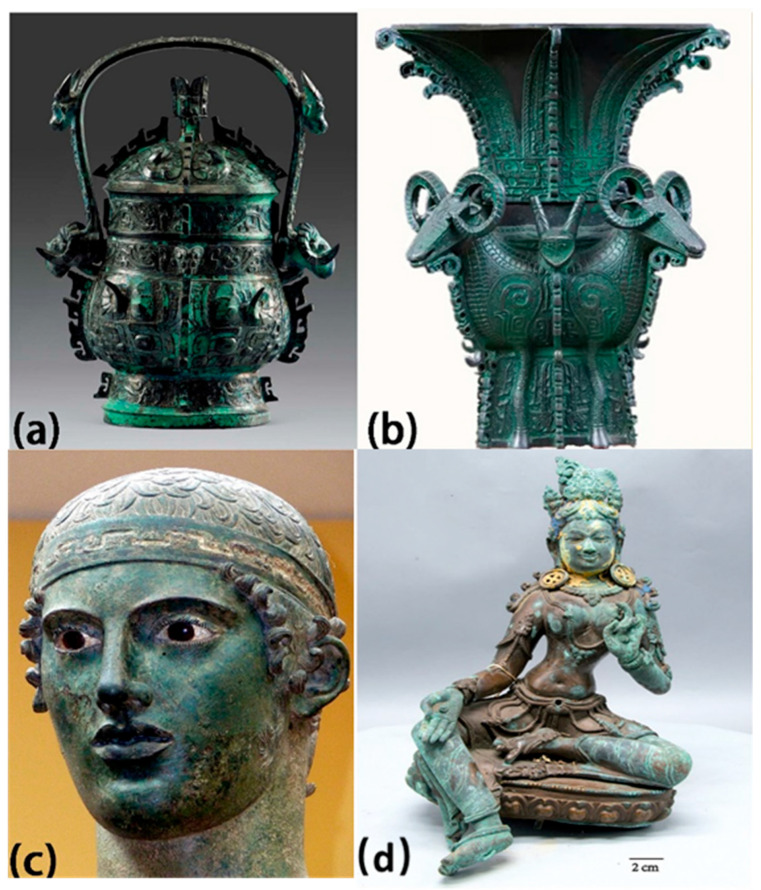
(**a**) Bronze wine vessel with handle Boge (1046~771 BC), Baoji Bronze Museum, Shaanxi Province, China; (**b**) The Square Zun with Four Goats (11th–10th century BC), National Museum of China; (**c**) Charioteer of Delphi (474 BC), Archeological Museum of Delphi; light green powdery corrosion products observed on different statues; (**d**) the gold-painted copper-based bodhisattva (Guanyin) in half lotus position dated to Qing Dynasty [8].

The Bronze Age (approximately 4000 BC to the beginning of the first century AD) was a time when metal objects first played an important role in human production and life, marking a significant stage in the development of human civilization [1]. Ancient bronze artifacts have unique shapes, exquisite decorations, and advanced casting techniques, possessing high artistic, historical, and cultural value, as well as scientific research value [2]. For example, bronze wine vessels with handle Boge (1046~771 BC, Figure 1a), were unearthed in 1980 at a cemetery in Baoji, China. There are two pieces, one large and one small, which are combined with the Zun to form a set of wine containers, and the decoration is elaborate and delicate, with a decorative style of ground pattern, shallow relief, high relief, and rounded carving; The Square Zun with Four Goats (11th–10th century BC, Figure 1b) was unearthed in 1938 in Hunan Province, which has four horned goat’s heads on each of the four corners of the shoulders, with the heads and necks of the goats protruding out of the vessel; Charioteer of Delphi (474 BC, Figure 1c) is one of the most famous Ancient Greek statues and one of the best preserved classical bronze casts, which depicts a charioteer in a chariot race as he presents his chariot and horses to the audience in recognition of his victory; The Sanxingdui site in Guanghan, Sichuan, first excavated in China in 1986, is known as the “source of the Yangtze River civilization” and it is one of the greatest archeological discoveries of the 20th century. Among the artifacts unearthed from Sanxingdui, the bronze mask group represented by the upright-eye masks is one of the most distinctive and spiritually and culturally significant artifacts.

Bronze artifacts carry important historical information and serve as powerful evidence for studying human history and ancient civilizations [3]. However, they were often buried underground for hundreds or even thousands of years, leading to varying degrees of damage and corrosion, with bronze disease being the most harmful type of specific corrosion phenomenon (Figure 1d) [4,5]. Bronze disease is particularly destructive due to its self-propagating electrochemical mechanism: the formation of porous copper chloride hydroxides (e.g., atacamite and paratacamite) generates large volume expansion, inducing mechanical stress that fractures the patina and exposes fresh metal to further attack; chloride ions (Cl^−^) are regenerated during hydrolysis of corrosion products (e.g., Cu_2_(OH)_3_Cl → 3Cu^2+^ + 3OH^−^ + Cl^−^), creating a self-sustaining loop that persists even in low-humidity environments. Once a bronze artifact is infected with bronze disease, its spread and development become difficult to control, which could result in the pitting and structural collapse, severely damaging its historical value [6,7]. Only if we have a comprehensive understanding of the alloy composition and structure of bronzes, and are familiar with the possible corrosion damage effects of various environmental factors, can we successfully use advanced characterization techniques to discover the corrosion damage mechanism of bronzes and assess the degree of corrosion damage when they are exposed to specific environments.

This article provides a systematic review of the corrosion behavior of bronze artifacts and advanced characterization techniques, summarizing the corrosion mechanisms of bronze artifacts and factors influencing corrosion such as their composition, structure, and external environment. It also details advanced analytical characterization techniques for corrosion products and mechanisms, and it concludes with prospects for future research on the corrosion and protection of bronze artifacts.

## 2. Corrosion of Bronze Artifacts

### 2.1. Corrosion Mechanisms of Bronze Artifacts

Bronze, a historic alloy, is composed primarily of copper and tin, and lead is often added for castability or other properties, as well as traces of iron, nickel, zinc, manganese, silicon, arsenic, and phosphorus, which are incidental impurities or intentional additions depending on the specific bronze. The basic process of bronze artifact corrosion involves environmental ions adsorbing on the surface of the artifact and undergoing chemical reactions with the metal atoms of the bronze artifact. If the reaction products are unstable, volatile, or decompose easily, the artifact will continue to corrode until it deteriorates. If the reaction products form a film on the surface of the artifact, creating a diffusion layer where metal atoms and environmental ions continuously generate compounds until the film can prevent further reaction, the artifact will no longer corrode. Research on the corrosion mechanisms of bronze artifacts mainly focuses on two aspects: observing the structure of the rust layers on bronze artifacts and investigating the mechanisms of corrosion, particularly exploring the causes of “bronze disease”. Table 1 summarizes some of the classic bronze artifacts that have endured corrosion, including their designation age, chemical composition, excavation site, burial environment, corrosion products, and corrosion characteristics. From the table, it can be roughly concluded that, due to the influence of various internal (material) and external (environment) factors, the types of corrosion products and corrosion characteristics on the surfaces of different kinds of bronze artifacts are quite different. This difference makes it particularly important to thoroughly characterize the corrosion behavior and analyze its corrosion mechanism to protect bronze artifacts effectively.

Microscopic studies of rust layer structures can help understand the chemical processes involved in the formation of corrosion products on bronze surfaces and establish models of interaction between bronze surfaces and interfaces with the local environment, identifying correlations between different bronze artifacts. Excavated bronze objects were typically covered with one or multiple layers of corrosion products, which are generally classified as harmless rust (Type I) and harmful rust (Type II) by Robbiola et al. (1998) [21]. Type I is characterized by even surfaces with excellent corrosion-resistant properties and can increase their artistic value. Type II structures are featured in coarse surfaces and the presence of high amounts of chloride on the internal layer or alloy interface, with localized corrosion phenomena or more generalized attacks due to the high dissolution rate of metal.

Harmful rust, on the other hand, can cause powdering and decay of artifacts, greatly reducing their lifespan. The powdery rust commonly found in bronze artifacts is mainly composed of Cu_2_(OH)_3_Cl, often referred to as “bronze disease”. This is a progressive corrosion induced by cuprous chloride, which accumulates within or on the surface of bronze until it reacts with water and oxygen to form cupric hydroxychloride, leading to volume expansion, internal physical pressure, and ultimately damage to the artifact. Bronze disease, characterized by powdery rust in bronze artifacts, is primarily composed of cupric hydroxychloride (Cu_2_(OH)_3_Cl). This destructive phenomenon originates from the catalytic oxidation of cuprous chloride (CuCl). The corrosion proceeds through the following self-accelerating steps:

CuCl formation: Buried bronze artifacts develop metastable CuCl layers (nantokite) through chloride-induced corrosion under anaerobic burial conditions.

Hydrolysis reaction: Upon exposure to moisture, CuCl hydrolyzes to form porous Cu_2_(OH)_3_Cl:

2CuCl + H_2_O + O_2_ → Cu_2_(OH)_3_Cl + HCl

Autocatalytic loop: This cycle sustains itself even with trace Cl^−^, driving continuous corrosion.

Acid regeneration: Released HCl further dissolves copper metal:

Cu + 2HCl → CuCl_2_ + H_2_

Redeposition of CuCl: Cu^2+^ ions react with Cl^−^ and Cu to regenerate CuCl:

Cu^2+^ + Cu + 2Cl^−^ → 2CuCl

The conversion from dense CuCl (ρ = 4.14 g/cm^3^) to porous Cu_2_(OH)_3_Cl (ρ = 3.75 g/cm^3^) causes ~250% volumetric expansion, generating internal stresses that fracture the patina and expose fresh metal. Moreover, CuCl acts as a permanent chlorine reservoir, enabling corrosion propagation without external Cl^−^ supply.

The structure of rust layers on bronze surfaces is closely related to the alloy type and preservation environment, and understanding corrosion forms and structures is beneficial for improving the research process and results of bronze corrosion mechanisms. Harmless rust typically denotes mineral phases forming dense, self-limiting oxide layers, such as cuprite (Cu_2_O), malachite (CuCO_3_·Cu(OH)_2_), or azurite (CuO). These compounds possess low solubility and compact crystalline structures, effectively blocking moisture and ion (Cl^−^, SO_4_^2−^) penetration to inhibit further corrosion of the underlying metal.

**Figure 2:**
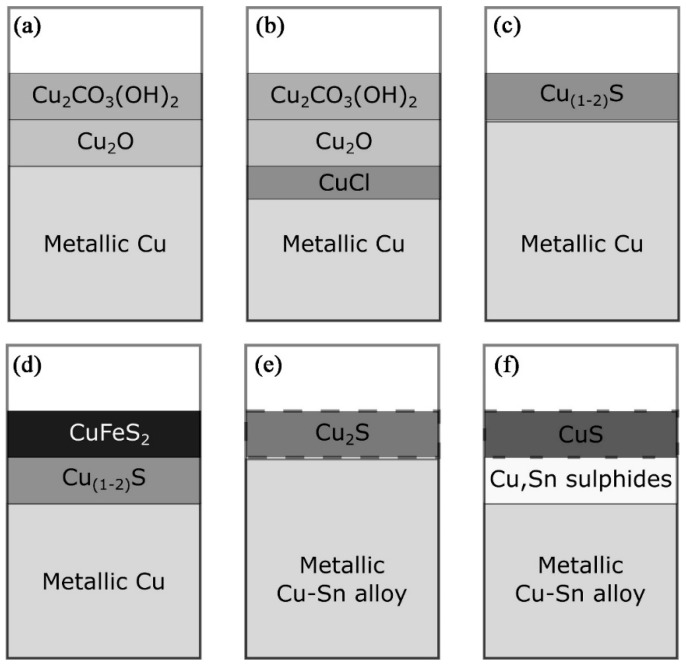
Schematic representation of the corrosion product layers formed on copper alloys in different burial environments: (**a**) malachite corrosion layers formed on top of cuprite in oxidizing burial environments; (**b**) a thin layer of copper chloride that would form in an oxidizing salt or marine environment; (**c**) porous copper sulfide layers often occur in sulfuric acid environments; (**d**) formation of a chalcopyrite (CuFeS_2_) layer on top of the silver sulfide layer if Fe^2+^ is present in the sulfuric acid environment; (**e**,**f**) characterize a complex corrosion sequence in which the oxygen corrosion products in an anaerobic environment are converted to Cu_2_S (in (**e**)) after further corrosion and conversion to make a CuS layer overlying the Cu, Sn sulfide layer [19].

Figure 2 shows the possible formation of corrosion product layers under different burial conditions [19]. In wet soil or oxidized moist environments, a layer of copper carbonate can form usually on the outer layer of copper oxide (Figure 2a) [21]; if in arid, marine, or other saline environments, copper chloride may form on the contact surfaces of metals and oxides (Figure 2b) since it is known that chloride ions (Cl^−^) may play a role in this case; in marine and estuarine environments, metals are attacked by sulfate-reducing bacteria and form corrosive layers of copper sulfides such as chalcopyrite (Cu_2_S) and/or covellite (CuS) (Figure 2c,d), and in some cases, it is possible to produce a chalcopyrite (CuFeS_2_) layer if there is sufficient dissolved iron in the burial environment (Figure 2d). The corrosion phenomena in the different environments described above are even more complex if we take alloying metals into account, such as tin (Sn), lead (Pb), and zinc (Zn): zinc in copper alloys is subject to corrosion and leaching; tin, because of its resistance to leaching, even when oxidized, is found relatively enriched on the surface of corroded bronze. High-tin bronze exhibits corrosion resistance due to the formation of a passive tin-rich layer on its surface during the corrosion process. During the corrosion of bronze, tin preferentially forms a dense layer of SnO_2_ (SnO_2_·nH_2_O). These SnO_2_ crystals have a rutile structure and exhibit high thermodynamic stability and low ionic diffusion rates. The open-circuit potential of SnO_2_ (~0.1 V vs. SHE) is lower than the dissolution potential of copper (~0.34 V), enabling the prioritized formation of a protective oxide layer that effectively blocks Cl^−^/H_2_O penetration. Micro-cells form between tin-enriched regions (cathodes) and adjacent copper phases (anodes) on the bronze surface. However, the current efficiency of these micro-cells is limited by the high impedance of SnO_2_, which inhibits the anodic dissolution of the copper matrix and slows the overall corrosion rate. Furthermore, the SnO_2_ layer physically isolates Cl^−^ from the copper substrate, thereby disrupting the catalytic cycle CuCl → Cu_2_(OH)_3_Cl and reducing the occurrence of bronze disease. However, during the sulfide corrosion process of bronze, complex copper and tin sulfides will form under the copper and tin corrosion layer (Figure 2e,f) [22]. Lead cannot form solid solution with copper (Cu) and tin (Sn) in copper alloys and can be easily oxidized once contacting with the environments. However, in a sulfidic environment, complex copper and tin sulfides are formed underneath the copper and sulfur corrosion layers (Figure 2e,f).

On the other hand, some progress has been made in the study of the corrosion mechanisms of bronze artifacts, but there is still considerable debate, such as the mechanism behind the formation of bronze disease, which remains inconclusive. The main corrosion mechanisms of bronze artifacts include the following: (1) **Intergranular corrosion**, which suggests that the corrosion mechanism of bronze artifacts is related to their inherent metallurgical characteristics. During the manufacturing process of ancient objects, repeated cycles of cold and hot mechanical processing and heat treatment may lead to impurities crystallizing along grain boundaries and segregation phenomena, possibly resulting in reduced mechanical properties and increased intergranular corrosion; (2) **Pitting corrosion**, where the main factors triggering the spread of powdery rust are humid and chloride-containing environments. The surface of bronze artifacts is uneven, and due to micro-potential differences, many micro-cells can form and undergo electrochemical reactions, leading to a series of oxidation reactions. However, this oxide film may be a mixture of various oxides, generally containing cracks, which further corrode in humid, chloride-containing environments, causing pitting corrosion on bronze artifacts; (3) **Selective corrosion**, where the migration of cations and other substances during localized corrosion processes in simulated corrosive media reveals that initially, the dissolution rate of tin is greater than that of copper, but after a period of corrosion, the dissolution rate of copper surpasses that of tin, making selective corrosion as the primary cause of bronze corrosion.

### 2.2. Influencing Factors on Corrosion Behavior of Bronze Artifacts

Corrosion of bronze artifacts is influenced by the casting process, alloy composition, alloy microstructure, and burial environments. Analyzing the corrosion layer structures and products of ancient bronzes is important for exploring corrosion behaviors and extracting archeological information.

#### 2.2.1. Influence of Composition and Structure of Bronze Artifacts

The corrosiveness of bronze is closely related to its composition and structure. Sn is the main alloying element in bronze artifacts, typically present in amounts ranging from 3% to 14% [18,23]. Within this range, the corrosion resistance of bronze artifacts increases with higher Sn content. Pb is usually present in bronze artifacts as a minor and auxiliary element. In Cu-Sn-Pb ternary alloys, lead exists as an independent phase in copper–tin alloys, dispersed in a “hollow spherical” granular form within the Cu-Sn solid solution matrix. Excavated bronze artifacts exhibit varying degrees of corrosion due to differences in tin and lead content [11,24]. Inhomogeneities in composition such as copper segregation, tin anti-segregation, free lead particles, uneven distribution, oxides, sulfides, etc., can lead to differences in composition between microscopically or macroscopically distinct areas of bronze artifacts [25]. These differences in composition, due to significant differences in electrochemical potential, can automatically trigger corrosion thermodynamically [26]. The addition of lead results in the formation of multiphase structures, with grain boundaries and phase boundaries typically having higher energy and being prone to corrosion initiation [15,27]. Corrosion microcells likely exist between different phases, accelerating corrosion or leading to localized corrosion. Current research on the impact of alloy composition and structure on the corrosion behavior of bronze alloys is not yet deep or systematic enough. To address the need for more systematic research, a multi-dimensional approach containing controlled alloy design, advanced microstructural characterization, corrosion testing, and quantitative modeling should be conducted to establish predictive relationships between alloy design, microstructure, and corrosion properties. It is necessary to precisely characterize the growth process of corrosion product films and elucidate the growth mechanism of corrosion product films under the coupling action of alloy elements and multiple elements, as well as the corrosion mechanism of bronze artifacts. Furthermore, researchers often overlook the impact of segregation phases or inclusions on the corrosion behavior of bronze alloys, and the micro-galvanic corrosion between segregation phases or inclusions and the bronze matrix is a significant factor promoting the corrosion of bronze alloys [10].

Marta Quaranta et al.’s study of bronze chariot decorations as well as horse harnesses and chimes from Tomb 27, excavated from an ancient burial pit at Liang Belt Village, Shaanxi Province, northwestern China [28]. Microstructural analysis demonstrated a cored dendritic structure with lead and copper oxide (Cu_2_O) globules in some samples. In addition, the researchers found that some samples showed extensive corrosion throughout the metallographic sections, and while the metallic core of the samples remained, other parts were completely mineralized. It is gratifying to note that the researchers found not only lead-based mineral products such as lead sulfate (angularite, PbSO_4_) and carbonate (cerussite, PbCO_3_), but also cuprite. The chemistry of the spherical network of big cuprite globules bulk cuprite was determined by Raman spectroscopy. Based on localized imaging maps of the cross-sections, the researchers found that the sample matrix contained unalloyed copper inclusions that replaced the eutectic in a pseudomorphic form or appeared spherical. Experiments have revealed a series of corrosion processes.

The experiments revealed a series of corrosion processes: lead oxide compounds (commonly known as PbO_X_) formed by the corrosion of metallic lead spheres may diffuse and migrate outward through the pores of the alloy and through the capillary channels formed during degradation, so that the lead spheres are left with voids as a result of corrosion [28]. At the same time, copper ions dissolved and corroded from the bronze alloy may oxidize, especially in close proximity to the surface of the alloy, and deposit in the voids left by the corrosion of the lead ball, forming a copper oxide layer. In this way, over time, copper oxides gradually and completely replace the original lead balls.

By analyzing the local corrosion behavior in the segregation zone of a low-Sn bronze alloy, the researchers found that the deviatoric zone has a high content of Sn [29]. However, the surface potential in this region is lower than that of the dendritic stem, leading to the formation of a micro-area galvanic couple, which induces the emergence of corrosion and accelerates the extension of corrosion. During the corrosion process, the rich Pb phase preferentially corroded as the anode and diffused towards the surroundings, while the rich Cu structure served as the cathode, and the corrosion products generated are mainly Cu_2_O and Cu_2_(OH)_3_Cl [30]. In comparison, the main corrosion products of the dendrites were Cu_2_O and SnO_2_, evolving into Cu(OH)_2_, Cu_2_(OH)_2_CO_3_, and Cu_2_(OH)_3_Cl, which were poorly protective for the local metal.

#### 2.2.2. The Influence of Environmental Factors

Environmental factors play a significant role in the corrosion behavior of bronze artifacts [31]. Cl^−^ is commonly present in soil-corrosive environments and is the main culprit behind the occurrence of bronze disease in bronze relics [20,32]. The main reasons for the exacerbation of bronze corrosion by Cl^−^ are the following: on one hand, Cl^−^ adsorbs on the surface of bronze artifacts, forms complexes with metal ions, and promotes metal dissolution; on the other hand, Cl^−^ can penetrate the passive film, accelerating the corrosion of the bronze matrix [12]. In a corrosive environment containing Cl^−^ and O_2_, bronze artifacts undergo electrochemical and chemical corrosion, where Cu elements undergo oxidation and react with OH^−^ to form Cu_2_O deposited on the surface of the bronze artifact [33]. Subsequently, some Cu_2_O will continue to react with Cl^−^ to form loose CuCl_2_·3Cu(OH)_2_ or Cu_2_(OH)_3_Cl, and some processes will also generate copper salts such as CuCl. The evolution of bronze disease involves a dynamic, multi-step reaction mechanism whereby the dominant pathway fluctuates according to environmental conditions. The formation of the key intermediate CuCl is inevitable. Electrochemical analysis and in situ Raman spectroscopy confirm that, in the presence of Cl^−^ ions, bronze corrosion preferentially proceeds via the following reaction:

Cu → Cu^+^ + e^−^ (anodic dissolution) → Cu^+^ + Cl^−^ → CuCl (nanocrystalline mineral).

In aqueous environments, CuCl evolves via a hydrolysis-oxidation pathway:

4CuCl + O_2_ + 2H_2_O → Cu_2_(OH)_3_Cl + Cu^2+^ + 3Cl^−^ + H^+^.

Among them, Cu_2_(OH)_3_Cl belongs to “powdery rust” and is a major product of “harmful rust,” which further leads to the corrosion and destruction of bronze artifacts. Temperature and humidity are two other important environmental factors that affect the corrosion of bronze artifacts. Temperature changes affect the solubility of gases or salts in the thin liquid film layer on the bronze alloy’s surface, thereby influencing the emergence of corrosion products on the surface of bronze alloys and their electrochemical corrosion rates. In addition to Cl^−^, temperature, and humidity, other factors, such as microorganisms in soil, acidic gases, such as CO_2_, O_2_, O_3_, and SO_2_ in water/air, and light exposure, also have varying degrees of impact on the corrosion of bronze artifacts [34,35]. The abundance of microorganisms and harmful substances in the soil can adhere to the surface of bronze artifacts, leading to their corrosion. The processes of microbiological corrosion and electrochemical corrosion are interrelated and mutually promote each other, accelerating the corrosion of bronze artifacts.

**Figure 3:**
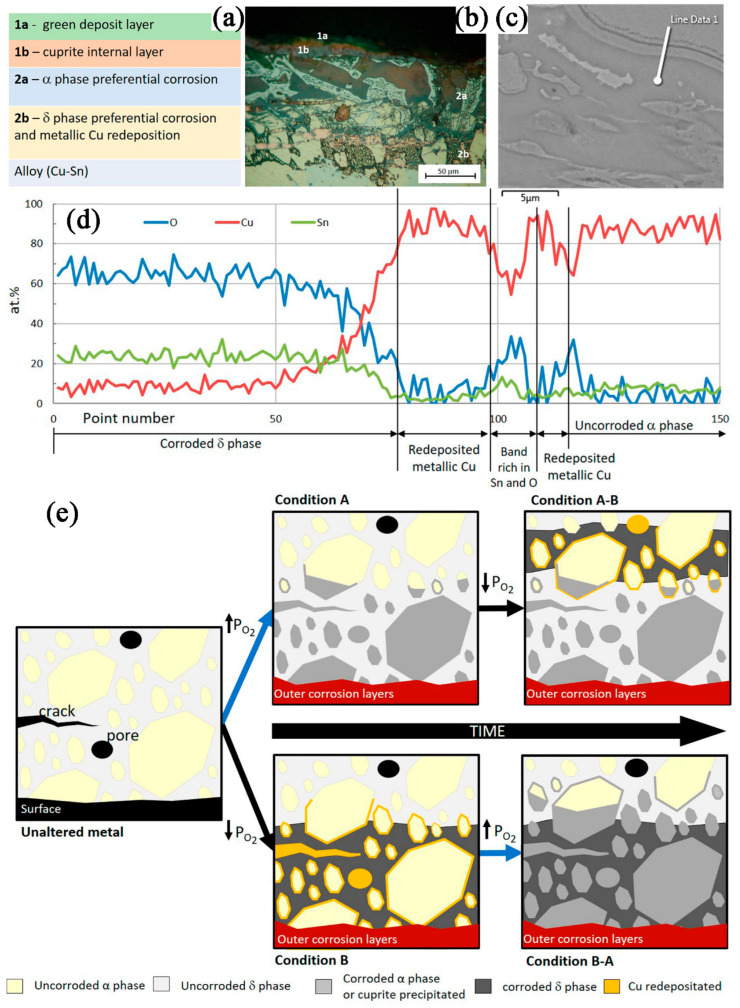
(**a**) Schematic representation of the different corrosion products layers; (**b**) cross-sectional OM image of the Coruche sample; (**c**) cross-sectional BSE-SEM image of the Famalicão sample; (**d**) cross-sectional line scan of Famalicão sample showed in (**c**); and (**e**) schematic representations of the microstructure of a bronze before corrosion and after corrosion under different conditions: **Condition A** (higher oxygen potential), **Condition B** (lower oxygen potentials) [16].

The layered corrosion structures of archeological high-tin bronze bells (>17 wt.%) (Figure 3) studied by Ana S. Saraiva in the presence of a large number of δ-phases can be classified into two main groups according to the precipitation mechanism: (1) the outer layer of corrosion product inclusions formed due to the metal ions leaching, and (2) the inner layer formed by redeposition of the corrosion products with no apparent alteration of the original morphology (Figure 3a) [16]. The outer corrosion layer includes two sublayers: (1a) a green deposit layer, which consists mainly of Cu[II] products, and (1b) a cuprite internal layer with red/orange in color due to the emergence of lots of copper oxides (Cu_2_O) and other Cu-Sn intermediates. Similarly, the internal corrosion layer contains two different sublayers: (2a) nearer to the surface, presenting preferential corrosion of the α phase (Cu rich), being the presence of δ phase without corrosion, and a deeper internal layer (2b) with preferential corrosion of the δ phase (richer in Sn) and precipitation of metallic copper, without α phase corrosion. The EDS line scan demonstrated the presence of a small band rich in O and Sn between two bands of precipitated copper (Figure 3d). The thickest Cu band may be associated with the redeposition of Cu after the oxidation of the δ phase. Due to the tin oxidation in the α grain border followed by its segregation to the grain boundaries and deposition in oxide form, a thin Cu band forms. Of the possible evolution of the different microstructural patterns summarized in Figure 3e, corrosion of Condition A is most likely to occur in archeological bronzes. Condition B may be related to the occurrence of δ-phase “networks” in high-tin bronzes. The chances of both conditions A-B and B-A occurring in the same artifact are not very high, unless there has been a fast-changing environment in which it is found. Condition B-A is even rarer as it implies a shift from less aerated and uncommon (Condition B) to more aerated (Condition A), which is related to the elevated oxygen potential at the corrosion front under burial conditions.

The corrosion process of bronzes is complex, and different types of corrosion can occur even in the same site. Bronzes excavated from the Sujia ridge cemetery in Hubei, China (the Sujia ridge cemetery dates from the early to mid Spring and Autumn period, ca. 770 B.C. to 550 B.C., show different corrosion products: malachite, cuprite, cerussite, azurite, and cassiterite [9]. However, a light greenish powder of trihydroxycopper chloride also appears on bronzes unearthed from different burials, which is a manifestation of bronze disease. Through analysis of the microstructure of the bronze artifacts and the burial conditions of the burials, the appearance of light greenish powder could be attributed to the high concentration of Cl^−^ in the burial soils. A survey of archeological contexts revealed that the uneven distribution of chlorine (Cl) within the same cemetery may be due to alternating drying and wetting conditions, which are caused by precipitation infiltrating different types of tombs in different areas. Pollutants produced by the current population in their daily lives may also be a contributing factor.

External environmental factors, such as acid-based atmosphere, oxygen content, active anions and cations, soil moisture, and the metabolism of some microorganisms, can lead to corrosion on bronze alloys, a fact that has been confirmed by numerous studies. Although the corrosion rate of objects buried in a long-term stable environment will gradually slow down, after being unearthed, the possibility of rusting and oxidization of bronzes exposed to oxygen-rich conditions for a long time is relatively high because of the large contact with the air [36]. The air with a great deal of water vapor, CO_2_, O_2_, etc., will be adsorbed in the loose rust layer on the surface of newly excavated bronzes, directly involved in the rusting process and promoting the formation of powdery rust on the surface of the artifacts. Acidic gases such as NO_2_ and SO_2_ present in the air have dissolving and acidifying effects, which have a significant impact on the corrosion of bronze artifacts. Light exposure can release photoelectrons from Cu_2_O on bronze artifacts, transforming into highly oxidizing photogenerated hole substances that can extract electrons from Cu atoms, leading to the corrosion of artifacts. Light exposure can also cause Cu_2_O to adsorb O_2_, further corroding the alloy components and thickening the surface rust layer. Therefore, museums have environmental quality requirements for the storage and display of their collections.

## 3. Characterization Techniques for Bronze Artifacts

To ensure that the shape of the bronzes and the composition and structure of their surface rust layers are detected and analyzed without damaging their surfaces, non-destructive testing is required. Non-destructive testing of excavated bronzes is an important part of the study of the phenomenon of corrosion of bronzes and the study of the protection of cultural relics [37]. Nowadays, the main equipment used for non-destructive testing of bronzes includes X-ray fluorescence (XRF), Laser Ablation Coupled to a Mass Quadrupole Spectrometry (LAMQS), X-ray tomography (CT), and Neutron diffraction [38].

### 3.1. X-Ray Fluorescence (XRF)

As a non-destructive means of elemental analysis, X-ray fluorescence (XRF) is used in a wide range of applications in materials science, environmental monitoring, geological exploration, archeology, and industrial quality control, as elements between Si and U can be detected, and elements as light as F can be detected by some portable instruments [39]. The XRF uses primary X-ray photon excitation of atoms in the substance to be measured, causing them to fluoresce for compositional analysis and chemical state studies, providing high-quality, non-destructive results [40,41]. It is important to note that the theoretical descriptions during the quantitative analyses were made under the assumption that the samples were perfectly flat and that each layer had similar densities. For artifacts with surface irregularities (e.g., corrosion pits and roughness) or density variations (e.g., patina layers and alloy inhomogeneities), several strategies are employed: matrix-matched standards (calibrate using reference materials with similar topography/composition to the artifact); multiple spot analyses (conduct 10–20 measurements across the artifact to average local inhomogeneities); computational corrections via fundamental parameters methods; and complementary techniques for density gradients. By integrating these strategies, XRF can provide practically robust quantification for bronze artifacts. However, artifacts are often inhomogeneous, irregularly shaped, or may be damaged. Therefore, to avoid the influence of the degree of damage to the artifacts on the XRF test results, it is necessary to take into account the inhomogeneity of the structure and some casting defects in the selection of sampling points [42]. In XRF analyses, the thicknesses investigated typically depend on the atoms that make up the material and on their absorption of X radiation. XRF estimates the thickness of materials by analyzing the absorption of X-rays and the intensity of fluorescence excitation within them. However, the accuracy of this method depends on the composition, density, and measurement conditions of the material.

**Figure 4:**
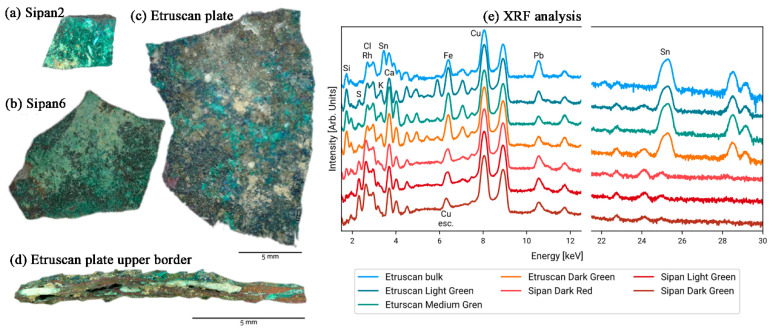
Visible images of the samples: (**a**) Sipan plate no. 2; (**b**) Sipan plate no. 6; (**c**) Etruscan plate; (**d**) Etruscan plate upper border with different layers; and (**e**) XRF spectra with the peaks of the chemical elements [43].

In 2011, researchers used X-ray fluorescence (XRF) technology to analyze several copper plates excavated in 2007 from an ancient tomb in the archeological area of Sipán to determine their composition (Figure 4a,b) [43]. Despite the copper-green surface of the samples, the presence of gold was still detectable in the analysis of the experimental data, leading the researcher to conclude that there may have been gold plating on the surface of these copper plate samples. The technique was also used in the material composition analysis of a bronze plate traceable to the Etruscan process (Figure 4c). All samples suffered severe corrosion degradation, with patinas of varying thicknesses and compositions on their surfaces (Figure 4d), showing varying degrees of green coloration, partly intermixed with blue coloration. In this case, the researcher, to determine the nature of the sample material, performed a single-point measurement using XRF (Figure 4e).

**Figure 5:**
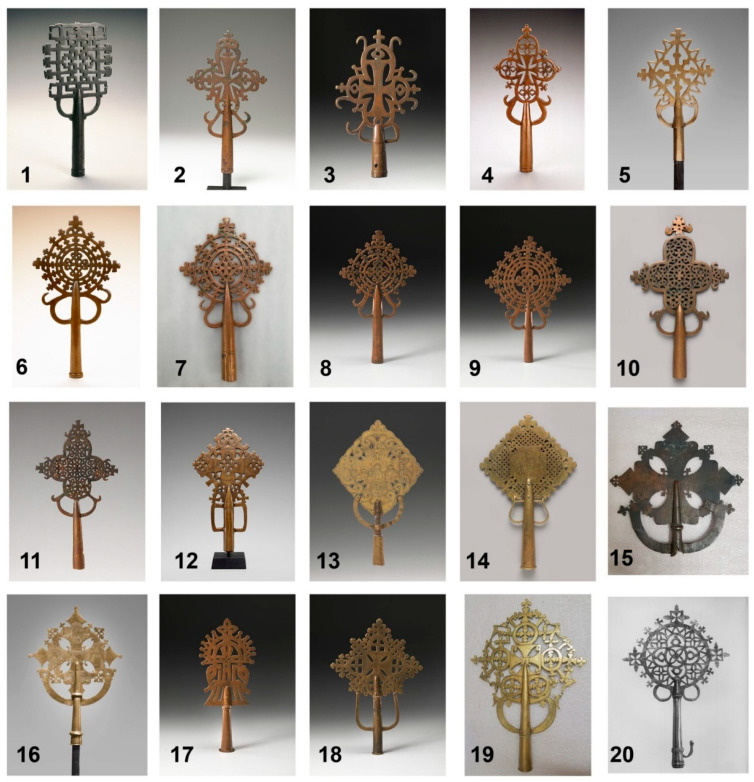
Processional crosses examined and analyzed [44].

The results of scanning microscopic XRF analysis of the materials used and the fabrication process of 20 cross samples indicate that XRF is of great practical value for the identification of ancient metalwork solder and castings, and for the preparation of restoration maps based on them (Figure 5) [44]. In addition, compositional analyses by XRF confirmed the pattern of alloy types for different styles of crosses from different eras: the predominant material for crosses dating as early as the twelfth to thirteenth centuries was identified as bronze, whereas those from the fourteenth to fifteenth centuries were identified as bronze, copper, or brass, and the predominant material for cross-making from the fifteenth century onwards was identified as brass or gunmetal. The above results can help the researcher to determine the period of making and production workshops of crosses of similar materials. The study found a clear preference for bronze over brass as the main material for crosses in the early period, which contrasts with the preference for brass for medieval Islamic and European metal ornaments. Differences in the materials used to make crosses from different periods may be due to changes in aesthetic preferences or in the availability of certain metals, and the disappearance of religious artifacts in the 16th century may have influenced the proportion of alloys used in the early surviving crosses. XRF can provide more solid data for further research on the fabrication process and determination of the material composition of metal objects.

### 3.2. Laser Ablation Coupled to a Mass Quadrupole Spectrometry (LAMQS)

Laser Ablation Coupled to a Mass Quadrupole Spectrometry (LAMQS) is an analytical technique developed by the Department of Physics at the University of Messina that allows the formation of ablation craters of varying depths on patterned surfaces (samples with spatially varying topography or composi-tional layers, e.g., bronze artifacts with corrosion layers, patina, or engineered microstructures) by controlling the interaction of the laser light with the target, thus enabling the analysis of atomic and molecular species under high vacuum conditions, with an accuracy of lead isotope ratios close to 0.1% [45]. For the experiments, the samples are placed in a high vacuum chamber on a holder that can be vertically and angularly offset, and photothermal ablation is induced by visible or infrared laser light. Layer-by-layer analysis can be achieved by fine-tuning the laser-material interaction to create controlled ablation craters.

**Figure 6:**
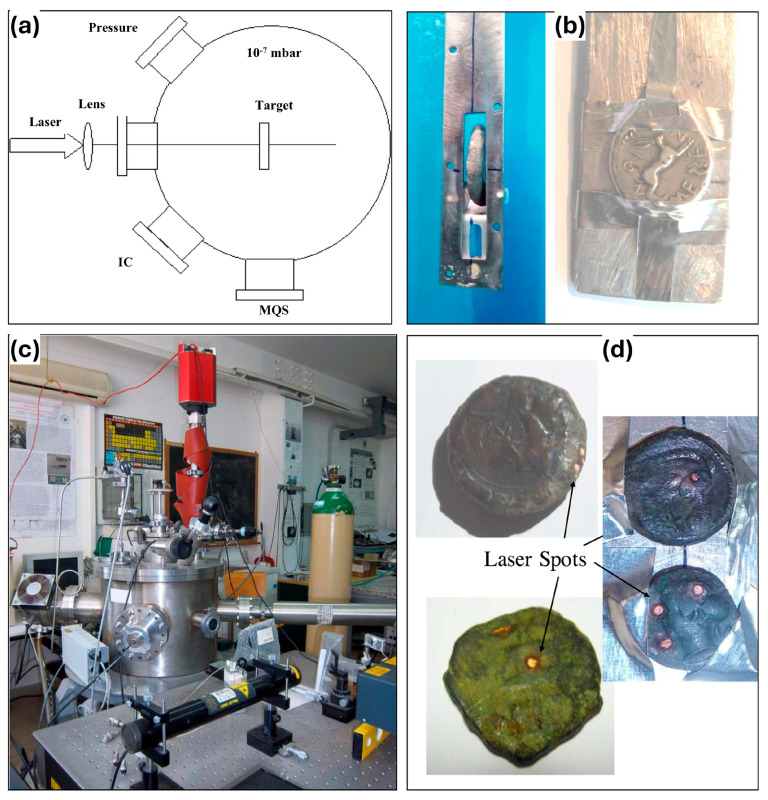
LAMQS equipment and sample: (**a**) diagram of the experimental setup; (**b**) photograph of the equipment; (**c**) photo of the coin holders, (**d**) photos of analyzed coins with laser spots [48].

For the experiments, the samples are placed in a high vacuum chamber on a holder that can be vertically and angularly offset, and photothermal ablation is induced by visible or infrared laser light. The mass spectrometer (MQS) is equipped with a continuous secondary electron multiplier (C-SEM) and an Ethernet interface for the detection of elements, compounds, and isotopes. The ion collector (IC) is positioned at an angle of 30° normal to the target to detect photons, electrons, and ions emitted from the laser-generated plasma during laser irradiation. (See Figure 6a,b for specific equipment layouts). The coin holder within the cavity (Figure 6c) is typically fixed at an angle of incidence of 0°. As shown in Figure 6d, the LAMQS removes the sample layer by layer by controlling the high-energy laser beam to create a pit of suitable depth on the sample surface [46,47]. This technique provides a very low sample removal rate and the smallest possible experimental area of the sample, which reasonably protects the integrity of the sample without compromising the results of the experiment.

**Figure 7:**
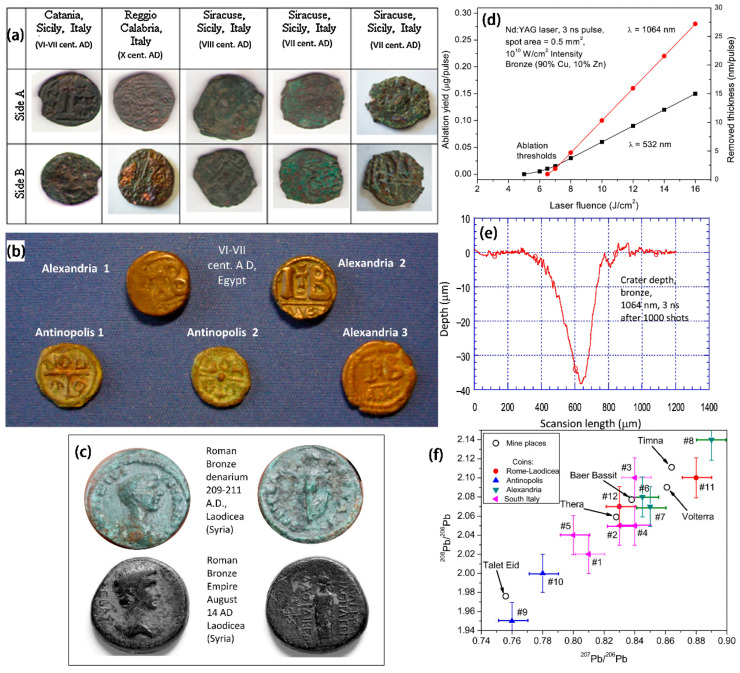
(**a**) Photographs of the three groups of bronze coins found in southern Italy (Reggio Calabria, Catania and Syracuse); (**b**) photographs of Egyptian bronze coins (Alexandria and Antinopolis); (**c**) photographs of bronze coins produced at the Roman and Syrian mints (Laodicean, Roman imperial period); (**d**) two wavelength calibration plots of ablated bronze artifacts (Cu = 90%, Zn = 10%); (**e**) typical depth laser profiles of bronze pits (1000 shots at 10 Hz repetition rate at 1064 nm); (**f**) typical lead isotope ratios of three groups of ancient bronze coins, compared with the geographic locations of the inferred mining areas obtained using the Brettscaife.net database [48].

LAMQS are often used to analyze the content of elements at different depths in ancient bronze coins. The three sets of bronze coins with different densities due to the different amounts of Pb were investigated in this way by L. Torrisia et al., as shown in Figure 7a–c [48]. A calibration study should be performed first in the LAMQS analysis to avoid the ablation rate as a function of laser fluence and number of laser exposures affecting the results. Typical calibration plots obtained by ablating a bronze substrate using two laser wavelengths (Figure 7d) reveal a composition of Cu = 90%, Zn = 10%: Cu = 90%, and Zn = 10%. In the experiment, the researchers evaluated the removal depth of each laser irradiation by ablation yield and obtained the desired removal depth by controlling the number of laser irradiations. With Figure 7e, we can see a typical crater depth profile for a bronze sample. With these methods, LAMQS was able to correlate the element concentration of each coin with the sample depth. By measuring the isotopes in different ancient bronze coins with LAMQS technology, the proportion of isotope mixing in different positions of some interesting elements can be determined, and then the chemical characteristics of ancient coins can be obtained. With Figure 7f we can see a comparison of the isotope ratios of 208Pb/206Pb and 207Pb/206Pb. The samples have significantly different Pb isotope ratios revealing that the samples are likely from different mining geographic locations and different historical periods. Moreover, the relative concentrations of elements measured by LAMQS are consistent with those measured by EDXRF. Thus, as a new technique to study ancient coins and any other archeological bronze metal artifacts, LAMQS can provide us with a powerful means of experimental detection and should be emphasized by researchers.

### 3.3. X-Ray Computed Tomography (CT)

As a non-invasive analytical technique, X-ray computed tomography (CT) can obtain morphological and physical information about the internal structure of materials on scales ranging from the tens of nanometers to meters [49,50]. The achievable resolution in X-ray computed tomography varies drastically depending on the equipment type, sample size, and imaging parameters. For the bronze corrosion studies, typical resolution of 0.5–50 μm can be achieved. CT technology utilizes the penetration ability of X-rays to collect a series of two-dimensional radiographs of a measured object from different angles (0–360°), in which the X-rays attenuate with the atomic number, composition, thickness, and density of the measured object. Subsequently, the computational reconstruction algorithm is performed to obtain the two-dimensional cross-section slices of the measured object, which offers the digital three-dimensional (3D) greyscale representation (tomographic image) of the internal structure of the measured object [51]. Due to the advantages of non-destructive methods, CT has great potential in cultural heritage research [52].

In 1977, Derek C.F. Harwood-Nash first utilized CT technology to scan a young female mummy, obtaining fine anatomical, Egyptological, and paleopathological information while avoiding damage to the cartonnage and the mummy itself [53]. Thereafter, numerous archeological artifacts have been researched via CT technology to establish conservation and restoration procedures and to examine and identify original pieces. Therefore, CT scanning plays an important role in obtaining the internal structure characteristics of artifacts.

**Figure 8:**
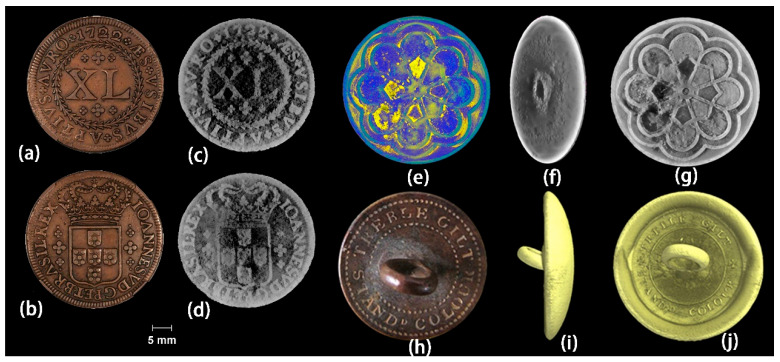
(**a**,**b**) Pictures of the coin C-04, (**c**,**d**) coronal views of the 3D models of coin C-04. (**e**–**g**) 3D models of sample B-01. (**h**) Internet picture of the button, (**i**,**j**) 3D models of sample B-02 [54].

S. Machadoa et al. used micro-CT technology to identify six unknown metallic artifact samples from the Antigo Museu Real restoration project in Rio de Janeiro, Brazil, including four coins and two ancient buttons, which were covered entirely by incrustation layers/corrosion crust [54]. The high-resolution CT scanning results gave a three-dimensional perspective and quantified the material loss (in percentage) of metallic artifacts caused by corrosion. Meanwhile, the value, year, life span, and mint location of the coins were identified, as well as the shape and trademark of the buttons. The comparison between the sample picture and the reconstructed 3D model results is shown in Figure 8. It can be seen that the sample C-04 (Figure 8a–d) was made of copper overall with a diameter of 36.00 mm and had the lowest material loss among the four coins (4.25%) caused by corrosion. The three-dimensional model of sample B-01 (Figure 8e–g) shows a different chemical composition at the top of the button that is less dense than the button base. This material is thought to be the decorative part of the button and is highlighted in yellow in Figure 8e. The loss of material due to corrosion for sample B-01 was 3.18%. The three-dimensional model of sample B-02 (Figure 8i,j) displayed a circular shape. The material loss due to corrosion for sample B-02 was 4.72%. The computed tomography results provide meaningful data on these historical artifacts, which are important for accurate and rapid diagnosis of their state of preservation, as well as an invaluable tool for preserving the morphology and internal structure of the collected artifacts.

**Figure 9:**
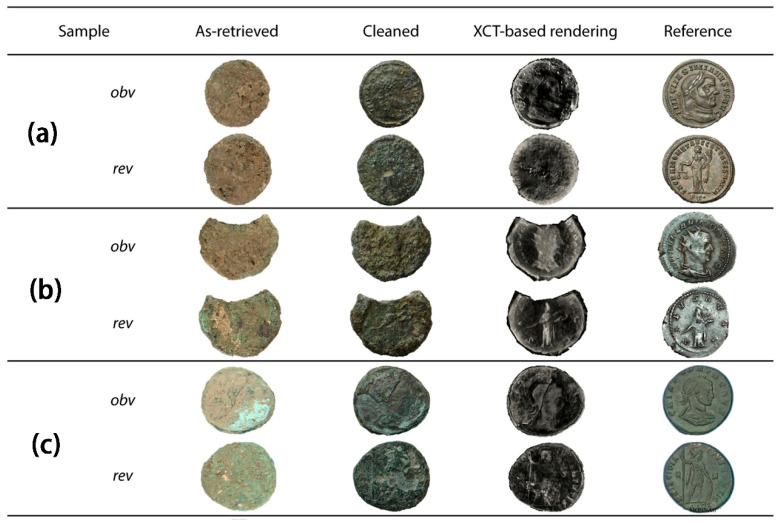
Three coins fully authenticated by CT. (**a**) coin a; (**b**) coin b; (**c**) coin c. The images of the coins correspond to the following: images extracted from the excavation site, after cleaning, 3D-rendered images based on tomographic reconstruction, and reference images from an online repository. Obverse (obv in the image) and reverse (rev in the image) are provided for each coin [55].

In order to fully analyze the ability of CT to identify Roman copper coins, Francesco Abate et al. compared the results of CT scanning with manual cleaning of corroded and soil-covered Roman copper coins [55]. The coins studied were found during archeological fieldwork in the vicinity of ancient Aquileia (Italy). Figure 9 presents pictures of copper coins retrieved from archeological sites, along with corresponding images collected after traditional cleaning and CT-based rendering. With regard to the **coin a** obverse, the information retrieved after cleaning was consistent with the results of the CT analysis, showing the outline of Maximianus and some letters. However, after cleaning the reverse of **coin a**, it was not possible to recognize traces of the female figure or legend found in the tomographic reconstruction. As for the **coin b** and **coin c**, their legibility was very low after physical cleaning. It can be found that the tomographic data provides more detailed information about the original surface, which is still retained within the corrosion layer, and therefore contributes to coin identification.

**Figure 10:**
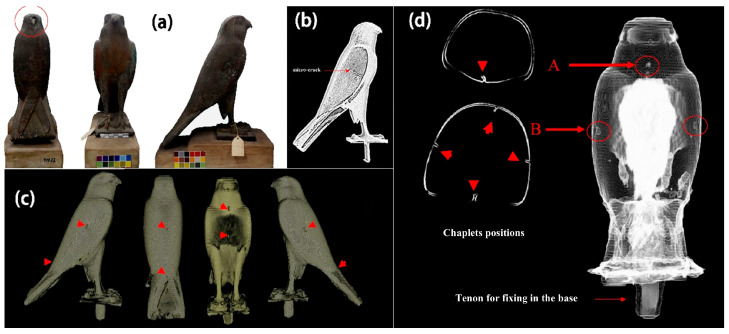
(**a**) shows god Horus bronze coffin from CEM collection, JE91922; (**b**) CT topography of the right-wing microfracture; (**c**) 3D CT images showing indications of the location of small flakes on the outer surface of the Falcon’s coffin reconstructed; (**d**) showing the location of the core flakes on the inner surface of the Falcon’s coffin, respectively. The letters A and B define two different flap positions [56].

MA Maher studied a hollow cast-bronze falcon statue of the god Horus standing on a metal pedestal in the collection of the Cairo Egyptian Museum (CEM) using multi-detector X-ray computed tomography [56]. Figure 10 shows photographic, 2D and 3D reconstructed CT images of the bronze coffin of the god Horus. The results showed that the bronze coffin was a monolithic hollow casting without any mechanical or thermal joints. Figure 10c shows 3D CT reconstructed images of all four sides of the casting and depicts the location of all sections of the outer surface of the falcon’s body. The 2D and 3D CT images (Figure 10d) provided the overall geometry of the coffin and showed all the components of the casting process. CT provided metric data on the wall thickness of the entire bronze casting and allowed for the observation of the different materials inside the casting and also provided insight into the characteristics of the casting process. Therefore, CT plays an important role in the inspection of bronze castings.

### 3.4. Neutron Tomography

Neutron diffraction usually refers to the Bragg diffraction of neutrons with a wavelength of about 1 Å as they pass through a crystalline material [13]. Neutrons are uncharged and highly penetrating, making them ideal for non-destructive examination of bronze artifacts [57,58]. Neutron imaging can therefore provide unprecedented three-dimensional information about bronze artifacts, allowing us to qualitatively, quantitatively, and visually analyze archeological materials and to obtain data on impurities, compositional variations, voids, and macrostructure [22,59].

**Figure 11:**
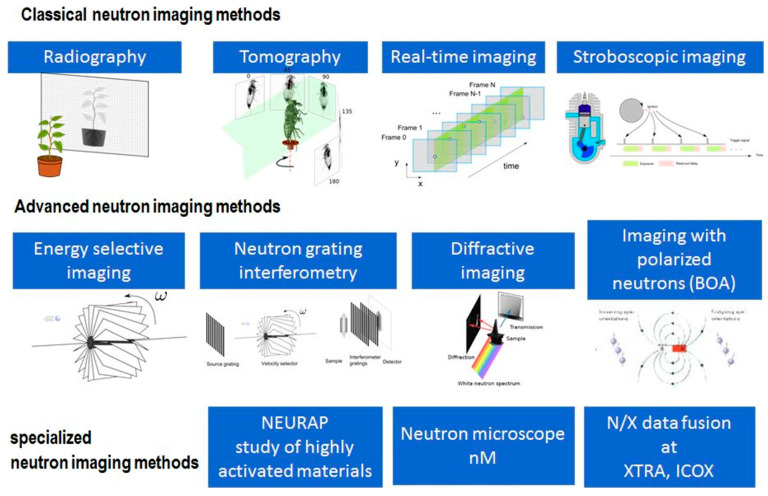
Overview of neutron diffraction methods [60].

Figure 11 illustrates a general overview of the research tools currently used in the field of Neutron diffraction methods, of which the radiographic mode is considered the simplest [60]. Other techniques are becoming more demanding to use and are only applied when justified. Nowadays, along with technological advances, computers are fully capable of reconstruction and visualization in a reasonable time acceptable to everyone, and so neutron tomography has developed into a standard tool. In the field of bronze artifacts research and in the study of material processing on different time scales, there will certainly be an increasing number of scholars interested in time-dependent studies. In all cases, the image quality is determined by the neutron dose applied. Figure 11 illustrates certain other methods, among which energy-selective imaging and diffraction imaging, as two completely non-invasive modalities, allow the study of crystal properties (grain size distribution, texture, internal strains, and crystalline phases) on a macroscopic scale with a resolution of up to about 50 µm.

**Figure 12:**
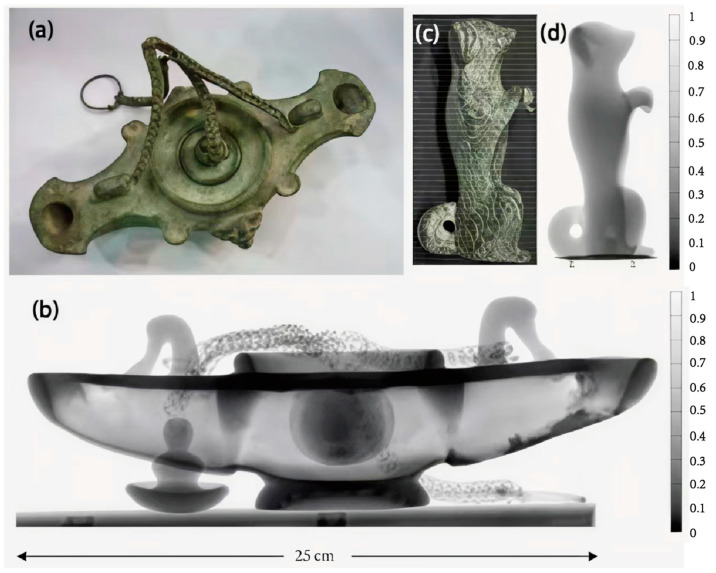
(**a**) Photograph of a late Roman hanging bronze lamp showing internal residues and (**b**) its two-dimensional neutron mosaic radiograph; (**c**) photograph of an ancient dog figurine and (**d**) its neutron radiograph. The transmittance scale on the neutron microscope goes from 0 to 1, with 0 indicating 0% neutron transmittance and 1 indicating 100% neutron transmittance [61].

Oak Ridge National Laboratory utilized the CG-1D prototype beamline at the High Flux Isotope Reactor to perform two- and three-dimensional neutron imaging inspections of hanging bronze lamps and bronze dog statues, and the researchers succeeded in obtaining an unprecedented amount of information, including fabrication details such as material properties, structure, performance, and technology of the samples [61]. Figure 12a presents a photograph of a late Roman hanging bronze lamp excavated from the site of Boscoreale, Italy, while Figure 12b is a 2D neutron mosaic radiograph of it. The 2D radiography clearly showed that the device was structurally stable except for minor corrosion on the exterior, and that there were no surface or subsurface cracks, voids, or other shortcomings. Moreover, the results of the 2D radiography tests clearly indicated the construction technique, texture, fuel residue, and combustion wear inside the lamp.

Figure 12c,d present photograph, neutron radiograph, and neutron transmission contour plots of a bronze dog figurine from the Joukowsky Institute’s collection (probably from the Roman period). The neutron radiograph data of the dog statue revealed defects such as voids inside the statue (Figure 12d). The internal structure of the statue contains clay and organic residues that attest to the use of lost wax technique casting.

**Figure 13:**
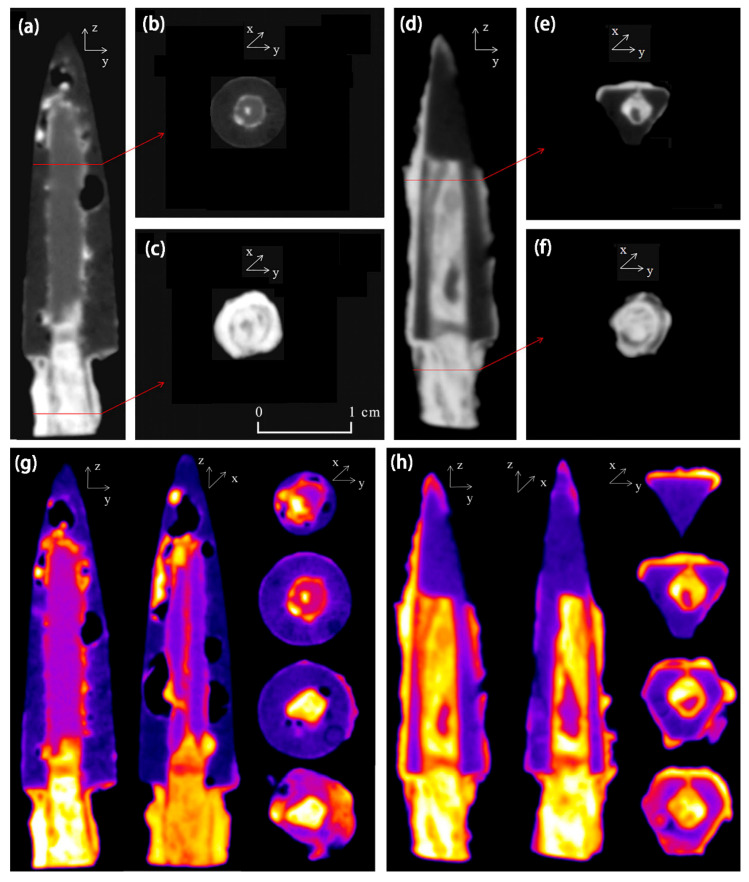
Neutron tomography of samples in different orientations: (**a**) center-axis section of sample A; (**b**,**c**) cross-section of sample A; (**d**) center-axis section of sample B; (**e**,**f**) cross-section of sample B; pseudo-color images of 3D slices: (**g**) sample A and (**h**) sample B [62].

Figure 13 shows an arrow made of different metals. The dark gray area in the image represents bronze, the light gray portion represents iron, and the white represents the corroded areas of bronze and iron [62]. The iron area within the arrowhead was straight and slender, with the boundary with the bronze clearly visible. The white area at the bottom of the arrow represents a cross-section of Tang bronze. In addition, Figure 13a shows the presence of structural defects in the bronze region, where porosity could have affected the mechanical properties of sample A, making it less resistant to external stresses. Mineral phase results from the samples measured using neutron diffraction indicated the occurrence of ferrite, cementite, and α-copper in the metallographic structure, from which it can be inferred that the main material used to make the Tang bronze was probably wrought iron and had a weak content of carbon. In addition, the researcher observed iron compounds consisting of goethite (α-FeOOH), hematite (Fe_2_O_3_), and magnetite (Fe_3_O_4_). Only cuprite (Cu_2_O) was found in the bronze arrowheads as the corrosion of bronze was not severe. Iron and bronze corrosion products often contain these compounds.

However, the main limitation of using the neutron diffraction method was the spatial resolution due to the influence of bulk measurements, and the experimental results often did not allow for the extraction of small amounts of corrosion products such as malachite [Cu_2_(OH)_2_CO_3_] and azurite [Cu_3_(OH)_2_(CO_3_)_2_] from the spectra. Due to two interrelated limitations—spatial resolution (in the range of 0.5–2mm, depending on collimation and detector geometry) and phase detection sensitivity—neutron diffraction struggles to resolve small quantities of corrosion products such as malachite (Cu_2_(CO_3_)(OH)_2_) and azurite (Cu_3_(CO_3_)_2_(OH)_2_). Neutron diffraction typically requires a phase to constitute more than 5% of the illuminated volume to produce detectable peaks. However, copper carbonates (malachite and azurite) often form subsurface layers or isolated microcrystalline patches that fall below this threshold. In fact, the presence of green and blue material on the surface of the arrowheads suggests a strong possibility that malachite and azurite participated in the corrosion process. Since chlorine-containing compounds like copper oxychloride [CuCl_2_-3Cu(OH)_2_-xH_2_O] and hematite [β-FeO (OH,Cl)] may accelerate corrosion, it is important to confirm their involvement as well.

**Figure 14:**
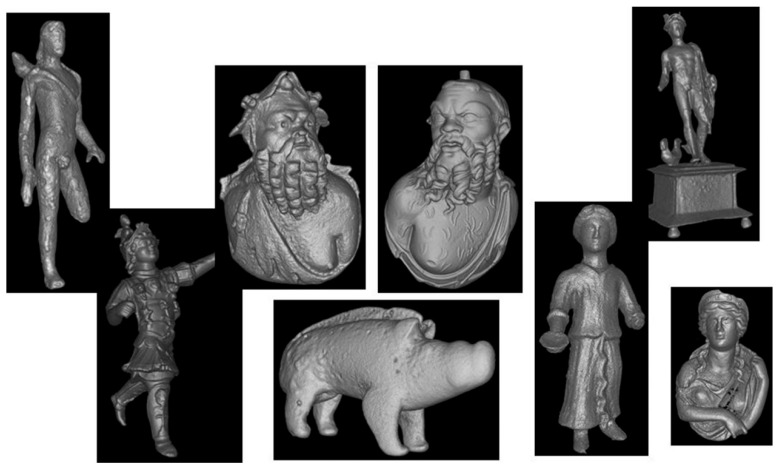
Some examples of tomographic studies of bronze artifacts from Swiss museums [60].

The Swiss National Museum and the University of Zurich have collaborated to launch a project for a systematic survey of Roman bronze sculptures excavated in the country [60]. Neutrons are more penetrating than X-rays, and about 200 samples were studied. Figure 14 shows an overview of some of the objects studied, all of which were around 2000 years old. The data allowed the discovery of material distribution, defects, casting residues, and information about past manufacturing processes. In some cases, corrosion and preservation conditions were also found.

The use of neutron diffraction techniques in the archeological study of bronzes was introduced above. Although XRF, CT, and neutron diffraction can all be used for the non-destructive examination of bronzes to observe cracks and pores, they all have certain limitations. For example, XRF is susceptible to interference from overlapping peaks of different elements. CT has a relatively large pixel size, with typical pixel intervals (spacing) typically ≥0.1 mm, and therefore detailed sample information is lost. Neutron diffraction is unreliable in determining single-cell parameters, and in situ testing at the excavation site is not possible due to bulky equipment and limited facilities capable of neutron diffraction.

### 3.5. Other Physical Methods

**Raman spectroscopy**, as a non-destructive method of chemical composition analysis, demonstrates its unique value in bronze studies [20,63]. It relies on inelastic scattering of materials by a laser light source to obtain specific information about the molecular vibrations of the material [17]. Thus, it is possible to deduce the material composition of the artifact and the likely manufacturing process. In the study of bronzes, the use of Raman spectroscopy can help researchers to identify a variety of inorganic and organic materials on the surface and within the artifacts, which can help to reveal the manufacturing process, historical usage, and the state of restoration and preservation of these artifacts.

**Figure 15:**
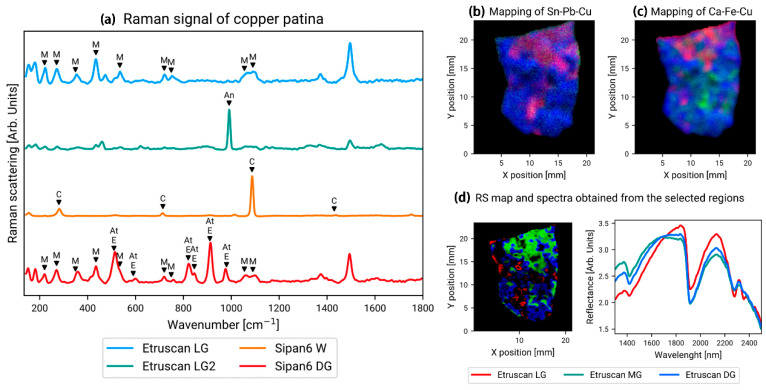
(**a**) Raman spectra of corrosion products of artifacts named Sipan and Etruscan with M, An, C, E, and At represent malachite, antlerite, calcite, eriochalcite, and atacamite, respectively. (**b**) Mapping showing the intensity of Sn (red), Pb (green), and Cu (blue) on the Etruscan plate; (**c**) mapping showing the intensity of Ca (red), Fe (green), and Cu (blue) on the Etruscan Plate; (**d**) the figure on the left is an RS mapping obtained from three Etruscan single-point surveys. The right figure is a spectrum obtained from a selected area of the RS mapping [43].

As shown in Figure 15a, Orsilli et al. used Raman spectroscopy to analyze the composition of corrosion products on two artifacts separately [43]. One artifact, named Sipan, is a collection of bronze medals found in 2007 in an ancient tomb in the archeological region of Shipan, Peru (South America), dating from the 2nd to 9th centuries AD. The other artifact, named Etruscan, was discovered during an archeological dig in central Italy and is believed to be an Etruscan artifact from the 2nd or 3rd century BC. With the help of Raman spectroscopy, we determined that the main components of the corrosive material qualitatively adhering to the surface of the Sipan sample were malachite, atacamite, and eriochalcite, while the calcite signal was believed to originate from an inclusion at the site where it was found. The malachite and antlerite were also detected in the Etruscan artifact.

**Fiber optics reflectance spectroscopy (FORS)** has been proven crucial in identifying “bronze disease” corrosion products on bronze surfaces thanks to its non-destructive, rapid, and non-invasive capabilities. Because of its high sensitivity and resolution, FORS can provide both qualitative and quantitative information about bronze corrosion products, including their chemical composition and distribution. Therefore, this technique enables rapid assessment of bronze disease progression and aids in devising appropriate protective measures [64]. The key spectral features of bronze disease (Cu_2_Cl(OH)_3_) include the strong absorption at ~740–780 nm assigned to Cu^2+^ d-d transitions in distorted octahedral Cl^−^ coordination, OH doublet at 1420 nm and 1930 nm derived from H_2_O/OH in the structure, and weak feature at 2180–2220 nm influenced by the Cl^−^ combination bands with OH. For the Malachite (Cu_2_CO_3_(OH)_2_), the key spectral features include that the Cu^2+^ peak shifts to ~800–820 nm due to the CO_3_^2−^ distortion, strong absorption at 2300–2350 nm assigned to CO_3_^2−^ peak, and no Cl^−^ signal can be detected. For the Antlerite (Cu_3_(SO_4_)(OH)_4_), the key spectral features include the Cu^2+^ strong absorption at ~700–720 nm and sulfate absorption at 2440–2480 nm. By leveraging these spectral discriminators, FORS provides rapid, non-destructive identification of bronze disease, enabling timely interventions like chloride extraction or inhibitor application.

Liu et al. used FORS to study the Ming Dynasty (1368–1644 AD) gold-plated copper cast “Vajrasattva” Bodhisattva statue, collected in the National Museum of China [5]. A total of 36 green powder corrosion spots on the surface of the bronze image were detected. FORS measurements show that these spots can be categorized into two different groups, with reference to their respective spectral characteristics. The first group, consisting of 17 samples, identified as copper trihydroxychloride (atacamite/clinoatacamite). The second group, consisting of 19 detection sites, exhibited different spectral features and was identified as chalcontonite. Chalcontonite was first identified in cultural heritage materials using FORS. FORS can provide a full scan of all corroded areas on complex artifacts in less than 30 min, accurately distinguishing between copper chloride and other green corrosion products such as malachite and chalcopyrite. In addition, because FORS uses fiber optic technology, it is not constrained by the size or shape of the object and is able to detect hard-to-reach areas such as internal surfaces and corners.

**Figure 16:**
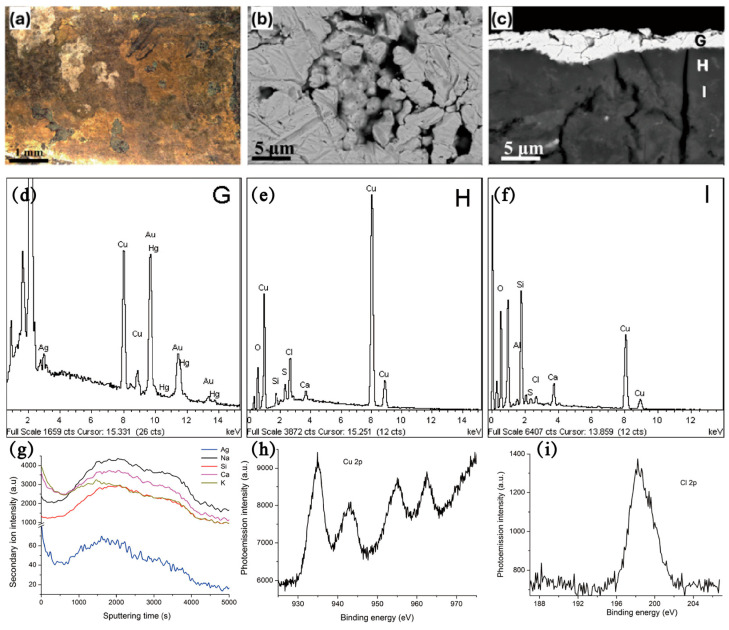
(**a**) OM image; (**b**) BSE images of the surface morphology of gold-plated brooches; (**c**) BSE images of cross-section microstructure of gold-plated brooches; (**d**–**f**) EDS spectra of the surface of the gold-plated brooches in (**c**) corresponding to G, H, I regions respectively; (**g**) ToF-SIMS depth profiles of the gold-plated brooches; (**h**,**i**) XPS spectra of (**h**) Cu 2p and (**i**) Cl 2p signals of the gold-plated brooches [72,73].

In addition to Raman and FORS, a variety of other techniques are employed for the characterization of bronzes, including optical microscopy (OM), scanning electron microscopy coupled with energy dispersive X-ray spectrometry (SEM+EDS) [65,66], scanning electrochemical cell microscopy [67], pulsed thermography [68], short wave infrared imaging [69], laser ablation–inductively coupled plasma–mass spectrometry [70], time-of-flight secondary ion mass spectrometry (ToF-SIMS), synchrotron radiation based methods [71], X-ray photoelectron spectroscopy (XPS) [14], and X-ray diffraction (XRD) (Figure 16) [72,73]. By combining these methods, the surface corrosion products of bronze can be accurately identified, providing crucial information for the restoration of cultural bronzes [73]. A comprehensive analysis of bronze corrosion requires methods that address spatial resolution, elemental sensitivity, chemical speciation, and depth profiling to be integrated. Due to the following trade-offs, no single method can address all aspects of bronze corrosion: (1) depth versus resolution—surface techniques (XPS) lack subsurface data, while bulk methods (XRD) miss nanoscale features, and (2) chemical versus structural data— chemical data is more sensitive than structural data. SEM-EDS shows element distribution but not bonding, a gap that is filled by XPS/XAS. By combining these techniques, conservators can create a chemically resolved 3D corrosion map, which is essential for accurate diagnosis and intervention.

## 4. Research Prospect

Nowadays, the related research on the corrosion behavior and corrosion mechanism of ancient bronzes are not rich enough and lack systematicity, and there are still limitations in the research on the influence of its own composition and external environmental factors on its corrosion mechanism and the diagnostic criteria of bronzes’ disease, and so on. For the excavation, treatment, and protection process of bronzes, the scientific problems and research outlook that need to be solved are as follows:

(1) Based on the development of material genome technology and machine learning methods [68], high-throughput experimental characterization techniques are used to rapidly screen new temporary sealing technologies that can be used at excavation sites to rapidly solidify excavated bronzes and provide effective corrosion protection. High-throughput experimental techniques can accelerate corrosion evaluation, making them a promising approach for developing new corrosion protection materials, such as anti-corrosion coatings and inhibitors. Özkan et al. explored the use of machine learning models to create an active learning loop for more efficient experimental discovery [74]. Their results are expected to support the development of faster inhibitor screening techniques that can capture the same higher solution electrochemical information on a shorter time-scale and more complex models that can leverage the link between the physicochemical nature of the inhibitor and its protective performance. By adapting these proven machine learning models, conservators can accelerate the discovery of reversible, non-toxic, and chloride-blocking sealants tailored to the micro-environments of archeological bronzes. The integration of artificial intelligence (AI) and machine learning (ML) into the study of bronze artifact corrosion is revolutionizing the field, enabling rapid data interpretation, predictive modeling, and conservation strategy optimization. Future work should focus on interdisciplinary collaboration between material scientists, archeologists, and AI specialists to harness these technologies responsibly.

(2) The existing non-destructive testing technologies are unable to perform quantitative analysis of the corrosion degree of bronzeware accurately, such as rust layer characteristics and corrosion extent. They can only provide a general detection of the composition of rust layers on bronze artifacts. Thus, we are eagerly looking to develop new techniques that are higher quality, more accurate, and more convenient for non-destructive testing of bronze patina at excavation sites. XRF primarily detects elements within 5–20 µm of the surface, failing to quantify subsurface chloride (Cl^−^) gradients critical for diagnosing “bronze disease” stages. Standard micro-CT achieves 1–5 µm resolution, insufficient to resolve nanoscale porosity (e.g., <500 nm cracks in CuCl layers) that dictate corrosion kinetics. Portable Raman Spectroscopy is limited to surface analysis (1–2 µm depth), failing to characterize sub-patina CuCl formation. Each laser pulse of Portable Raman Spectroscopy removes 0.1–1 µg of material, altering surface morphology and limiting repeated measurements on delicate artifacts. These limitations highlight the need for next-generation hybrid systems integrating deeper penetration, higher resolution, and non-contact operation for accurate field quantification of bronze corrosion.

(3) Nowadays, the quantitative classification standard for determining the level of bronze disease is still not uniform, and researchers are actively looking for new non-destructive and micro-destructive rapid analysis techniques that can be used in the excavation site to accurately characterize the surface corrosion products of bronzes and their physical and chemical information to establish a system of quantitative standards and diagnostic methods for bronze disease. A standardized multi-dimensional classification framework based on chemical composition, morphological features, corrosion kinetics, and environmental risk factors can be proposed. The chemical composition can contain chloride content (Cl^−^), sulfur coexistence, and corrosion product ratio, which can be detected by XRF, Raman Spectroscopy, and FTIR. The morphological features can include porosity, surface roughness, and layer delamination, which can be detected by Micro-CT and optical profilometry. The corrosion kinetics and electrochemical parameters detected by in situ electrochemical sensors can include the corrosion current density, critical relative humidity, and diffusion coefficient.

(4) Research on the effects of the collection environments (temperature, humidity, light, H_2_O, CO_2_, O_2_, Cl^−^, microorganisms, sound, as well as trace SO_2_, NO_2_, CO, O_3_, etc.) on the corrosion behavior of bronzes is lacking. In order to monitor in real time the corrosion evolution process of bronzes, and to explain the corrosion deterioration mechanism and the failure mechanism of long-term sealing materials, a specification of the in situ electrochemical test should be established in collection environments.

(5) Standardized data collection requirements and specifications should be developed, and an all-encompassing database of bronze burial environmental factors—bronze composition and disease grades—as well as a database of bronze descaling and protection materials and application cases should be established.

## Figures and Tables

**Table 1:** Summary of some classic bronze artifacts enduring corrosion.

Artifact	Age	Chemical Composition	Excavation Site	Burial Environment	Corrosion Products	Corrosion Characteristics	Ref.
M79 Dings and Guis; M88 Fu; M85 Fu; M102 Li; M26 Hu; M107 Hu	Early Spring and Autumn Period to Mid Spring and Autumn Period (~770 B.C. to 550 B.C.)	Cu-Sn-Pb	Southern part of Sujialong Cemetery (Hubei, China)	Soil	Copper trihydroxychloride/cassiterite, cuprite, and tenorite	With/without powdery rust	[9]
Wu Zhou coins	Han Dynasty of China	Cu (84.8–85.4%)-Sn (3.3–6.1%)-Pb (4.7–6.4%)-Sb (2.6–2.9%)	Zhongguan minting site, 25 km west from Xi’an city, China	Soil	Cu_2_(OH)_3_Cl, Cu_3_(CO_3_)_2_(OH)_2_, Cu_2_(OH)_2_CO_3_, and Pb_3_O_4_	Rough surface cracks, pits, and multicolor patina	[10]
Ancient Chinese bronze	Warring States Period of China (453–221 BCE)	Cu (66.88%)-Sn (22.92%)-Pb (10.2%)	No. 14 tomb of Wangjiachong, Huangzhou district, Huanggang city, Hubei province, China	Wet and acidic burial conditions	Cu_2_(CO_3_)(OH)_2_, SnO_2_, PbO	Harmless rust	[11]
Roman leaded-bronzes	4th century BCE to the 2nd century CE	Cu (75.61–91.97%)-Sn (3.87–11.47%)-Pb (0.95–19.80%)	“Punta del Serrone” area (Apulia), the Sicilian channel (Sicily) and the Arburese coast (Sardinia)	Seawater	MgSn(OH)_6_, CaCO_3_, PbCO_3_, Cu_2_S, MgS, Cu_2_O, Mg(OH)_2_, Cu_2_S, Cu_1.78_S	Bronze disease	[12]
Bimetal bronze sword	Warring States period (476–221 BC)	Cu-Sn (16%)-Pb (7%)	Hunan Province China	Beside the decomposed bone in the tomb	Malachite (Cu_2_CO_3_(OH)_2_), cuprite, cassiterite, quartz, pyromorphite, libethenite, goethite, phosphogartrellite, romarchite, anarkite, lepidocrocite	Selective corrosion	[13]
Sardinian bronze axe	Iron Age (10th century BC)	Cu (86.9–95.4t%)-Sn (2.8–6.9%)-As (0.6–1.3%)	Motya (Sicily, Italy)	Soil	Cuprite (Cu_2_O), cassiterite (SnO_2_), laurionite (PbClOH), cerussite (PbCO_3_), litharge (PbO), anglesite (PbSO_4_) and plumbonacrite (Pb_5_O(OH)_2_(CO_3_)_3_)	Selective corrosion	[14]
Bronze fragment samples (Fang, Mou, Bi Earcup, Jiaodou)	Western Han of China	Cu (76.1%)-Sn (8.2%)-Pb (5.2%)	Zhong county, Yunyang county, Shizhu county and Fuling county of Chongqing, China	From tombs near the Yangtze River	pyromorphite, mimetite, malachite, azurite (Cu_3_(CO_3_)_2_(OH)_2_), cuprite, cerussite, Cu_2_CO_3_(OH)_2_, Cu_3_(CO_3_)_2_(OH)_2_, malachite, cerussite, Pb_5_(AsO_4_)_3_Cl, Pb_5_(PO_4_)_3_Cl, mimetite-pyromorphite	Selective corrosion	[15]
High-tin bronzes (bells from foundry pits)	Thirteenth to the nineteenth centuries	Cu (72.7–79.3%)-Sn (19.4–27.3%)-Pb (<3%)	Portuguese	Soil	SnO_2_·xH_2_O	Selective corrosion	[16]
Gilded bronze statue”Vajrasattva Bodhisattva”	Ming dynasty of China (A. D. 1368–1644)	-	National Museum of China	Atmospheric environment	Copper trihydroxychloride (atacamite/clinoatacamite) and chalconatronite	Powdery corrosion	[5]
Ancient Roman coins (antoniniani)	260 to 270 CE	Cu (71–91%)-Sn (0–9%)-Pb (<4–23%)-Ag (2–17%)	Hoard of Cumae (Campania Region, Southern Italy)	Soil	Cuprite, malachite, cerussite, copper chloride, silver chloride	Bronze disease	[17]
Corinthian and Illyrian helmets	Archaic period	Cu-Sn (<14%)	Greece	Soil	Malachite, azurite, cuprite	Powdery corrosion	[18]
Roman copper alloy coins	From Vespasian (AD 69–79) to Marcus Aurelius (AD 161–180)	Cu-Sn-Pb-As-Ag	Netherlands and Switzerland	Alternations or progressive environment	Iron oxides, tin-oxide, copper sulfides, copper and copper-iron sulfides, malachite	Selective corrosion	[19]
Roman Empire Antoninian coins	Third century AD (Antonine age)	Cu-Sn-Pb-Ag	Fontanamare Discovery (Sardinia Coast, Italy)	Submarine conditions	Atacamite (Cu_2_Cl(OH)_3_), botallackite (Cu_2_(OH)_3_Cl), cuprite (Cu_2_O), cassiterite (SnO_2_), lead carbonates cerussite (PbCO_3_) and the lead oxide, lead chloro-carbonate phosgenite (PbCl)_2_CO_3_), Cu_2_S (chalcocite), CuS (covellite)	Bronze disease, microbial-induced corrosion (MIC)	[20]

## Data Availability

No new data were created or analyzed in this study. Data sharing is not applicable to this article.
